# Effects of Digital Sleep Interventions on Sleep Among College Students and Young Adults: Systematic Review and Meta-Analysis

**DOI:** 10.2196/69657

**Published:** 2025-05-12

**Authors:** Yi-An Lu, Hui-Chen Lin, Pei-Shan Tsai

**Affiliations:** 1 School of Nursing College of Nursing Taipei Medical University Taipei Taiwan; 2 Research Center in Nursing Clinical Practice Wan Fang Hospital, Taipei Medical University Taipei Taiwan; 3 Department of Nursing and Center for Nursing and Healthcare Research in Clinical Practice Application Wan Fang Hospital, Taipei Medical University Taipei Taiwan; 4 Research Center of Sleep Medicine Taipei Medical University Hospital Taipei Taiwan

**Keywords:** emerging adults, insomnia, sleep quality, technology-assisted sleep interventions, undergraduate students

## Abstract

**Background:**

College students and young adults (18-25 years) frequently experience poor sleep quality, with insomnia being particularly prevalent among this population. Given the widespread use of digital devices in the modern world, electronic device–based sleep interventions present a promising solution for improving sleep outcomes. However, their effects in this population remain underexplored.

**Objective:**

We aimed to synthesize current evidence on the effectiveness of electronic device–based sleep interventions in enhancing sleep outcomes among college students and young adults.

**Methods:**

In total, 5 electronic databases (PubMed, CINAHL, Cochrane Library, Embase, and Web of Science) were searched to identify randomized controlled trials on digital sleep interventions. Sleep interventions, including cognitive behavioral therapy for insomnia, mindfulness, and sleep education programs delivered via web-based platforms or mobile apps, were evaluated for their effects on sleep quality, sleep parameters, and insomnia severity. Pooled estimates of postintervention and follow-up effects were calculated using Hedges *g* and 95% CIs under a random-effects model. Heterogeneity was assessed with *I*^2^ statistics, and moderator and meta-regression analyses were performed to explore sources of heterogeneity. Evidence quality was evaluated using the Grading of Recommendations Assessment, Development, and Evaluations framework.

**Results:**

This study included 13 studies involving 5251 participants. Digital sleep interventions significantly improved sleep quality (Hedges *g*=−1.25, 95% CI −1.83 to −0.66; *I*^2^=97%), sleep efficiency (Hedges *g*=0.62, 95% CI 0.18-1.05; *I*^2^=60%), insomnia severity (Hedges *g*=−4.08, 95% CI −5.14 to −3.02; *I*^2^=99%), dysfunctional beliefs and attitudes about sleep (Hedges *g*=−1.54, 95% CI −3.33 to −0.99; *I*^2^=85%), sleep hygiene (Hedges *g*=−0.19, 95% CI −0.34 to −0.03; *I*^2^=0%), and sleep knowledge (Hedges *g*=−0.27, 95% CI 0.09-0.45; *I*^2^=0%). The follow-up effects were significant for sleep quality (Hedges *g*=−0.53, 95% CI −0.96 to −0.11; *I*^2^=78%) and insomnia severity (Hedges *g*=−2.65, 95% CI −3.89 to −1.41; *I*^2^=99%). Moderator analyses revealed several significant sources of heterogeneity in the meta-analysis examining the effects of digital sleep interventions on sleep outcomes. Variability in sleep quality was influenced by the sleep assessment tool (*P*<.001), intervention type and duration (*P*=.001), therapist guidance (*P*<.001), delivery mode (*P*=.002), history of insomnia (*P*<.001), and the use of intention-to-treat analysis (*P*=.001). Heterogeneity in insomnia severity was primarily attributed to differences in the sleep assessment tool (*P*<.001), while the effect size on sleep efficiency varied based on intervention duration (*P*=.02). The evidence quality ranged from moderate to very low certainty across measured outcomes.

**Conclusions:**

Digital sleep interventions are effective in improving sleep quality and reducing insomnia severity, with moderate effects on dysfunctional beliefs and attitudes about sleep, sleep hygiene, and sleep knowledge. These interventions offer a viable approach to managing sleep problems in college students and young adults.

**Trial Registration:**

PROSPERO CRD42024595126; https://www.crd.york.ac.uk/PROSPERO/view/CRD42024595126

## Introduction

### Background

College students and young adults aged between 18 and 25 years frequently experience poor sleep quality and insufficient sleep duration [1,2] accompanied by a high prevalence of insomnia. Studies have reported that 18.5% of college students have insomnia, whereas 22.6% of young adults experience difficulty falling asleep; these rates are higher than those observed in the general population [3,4]. Adequate sleep is essential for maintaining overall health, cognitive functioning, and overall well-being in college students and young adults [5,6]. Although the recommended sleep duration for individuals aged 18 to 25 years is 7 to 9 hours per night [7], a study indicated that 30% to 50% of college students and young adults fail to meet this recommendation [8].

Short sleep duration is associated with numerous adverse outcomes in college students and young adults. These include poor dietary habits, obesity [9], and an increased risk of mental health problems (eg, depression, suicidal behavior, and substance abuse) [10-13]. Furthermore, insufficient sleep adversely affects academic performance and may lead to a need for prolonged years of study [14]. Poor sleep in college students and young adults can also contribute to long-term sleep disturbances that may persist into adulthood [15].

Meta-analyses conducted by Friedrich and Schlarb [16] and Saruhanjan et al [17] have indicated that psychological interventions exert a substantial moderate-to-large effect on sleep-related outcomes in college students. In recent years, rapid technological advancements and the widespread adoption of electronic media have drawn attention to the relationship between electronic media use and sleep. A study indicated that college students and young adults spend an average of 316 minutes per week using electronic media, such as smartphones, tablets, emails, and video platforms [18]. This demographic also engages with the internet more frequently than other age groups, rendering it a central aspect of their daily routines [19]. Internet-based interventions have become a practical and promising solution to sleep problems, offering a form of therapy that individuals can access at their preferred time and location [20]. Although face-to-face interventions are highly effective [21], they are often costly, time-intensive, and impractical in many colleges or other settings [22,23]. By contrast, electronic device–based interventions offer numerous advantages, including convenience, affordability, privacy, ease of distribution, and consistent content delivery [24] These interventions have demonstrated comparable effectiveness to face-to-face methods in addressing sleep difficulties among college students and young adults [17].

Several studies have systematically reviewed the effectiveness of digital psychological interventions for college students [25-27]. However, these reviews did not specifically evaluate sleep-related outcomes. One review [26] included only 2 studies on sleep, neither of which focused on sleep as a primary outcome, thereby limiting the specificity of the analysis regarding sleep-related interventions. Furthermore, the effects of electronic device–based sleep interventions on sleep outcomes in college students remain underexplored. In total, 4 systematic reviews have examined the effectiveness of digital cognitive behavioral therapy for insomnia (CBT-i) [28-31]. Among these reviews, 2 [30,31] included college students. Tsai et al [30] reviewed 4 studies, of which 1 included adolescents and 3 focused on college students. However, certain forms of digital CBT-i, such as interactive therapy and CBT-i delivered using mobile apps, were not analyzed, which considerably limits the applicability of their findings. In addition, Zhang et al [31] analyzed digital sleep interventions in individuals aged 18 years and older with insomnia or poor sleep quality. Although the review included diverse populations, only 2 studies specifically focused on college students. The other 2 reviews [28,29] focused on a broad range of participants aged 18 years and older, including adults and patients with sleep disorders. In addition, these reviews exclusively relied on subjective sleep outcome measures, not including objective data in their analyses [28,29]. Although these studies have provided strong evidence indicating improvements in sleep quality and insomnia symptoms, the heterogeneity in the characteristics of their participants may have affected the internal validity of their results, thereby limiting the generalizability of their findings.

Research indicates that psychological interventions can substantially improve sleep quality and mental health in college students. However, the impact of digital interventions on sleep among college students and young adults remains underexplored. Given the high prevalence of sleep difficulties in this population and their widespread use of digital devices and mobile apps, investigating the effectiveness of digital sleep interventions is both relevant and crucial. This systematic review and meta-analysis focused on college students and young adults aged 18 to 25 years [32]; this is a critical developmental stage often marked by poor sleep health [33]. Importantly, this review addressed several limitations in literature by focusing solely on sleep-related outcomes in college students and young adults. Specifically, we examined both immediate and longer-term effects of digital sleep interventions on subjective and objective sleep measures. By including a wide variety of digital modalities, this review offers clearer insight into how technology-assisted sleep interventions can help improve sleep in this population.

### Objectives

This systematic review and meta-analysis thus aimed to synthesize and quantitatively evaluate current evidence regarding the effectiveness of electronic device–based sleep interventions in enhancing sleep quality, reducing insomnia severity, and improving sleep outcomes among individuals aged 18 to 25 years, in comparison with control conditions.

## Methods

### Study Design

This systematic review and meta-analysis of randomized controlled trials (RCTs) investigated the effects of digital sleep interventions on college students and young adults aged 18 to 25 years. The included interventions were delivered via web-based platforms or mobile apps. The review was conducted in accordance with the PRISMA (Preferred Reporting Items for Systematic Reviews and Meta-Analyses) guidelines [34], which are detailed in Multimedia Appendix 1. The review protocol was registered with PROSPERO (CRD42024595126).

### Data Sources and Search Strategy

Two reviewers (YAL and HCL) worked collaboratively to conduct a comprehensive search across 5 electronic databases, namely PubMed, CINAHL, Cochrane Library, Embase, and Web of Science, to identify relevant studies published from each database’s inception to October 2024. The search strategy involved the use of the following keywords: “(college OR university AND students OR young adults) AND (digital OR sleep AND intervention OR internet OR web-based OR online OR email OR mobile OR smartphone OR virtual reality) AND (sleep OR sleep quality OR insomnia) AND (randomized controlled trial).” Details regarding the search strategies are provided in Table S1 in Multimedia Appendix 2. In addition, the reference lists of related articles were manually screened for further eligible studies. No restrictions regarding the language of publication were applied.

### Inclusion and Exclusion Criteria

In this systematic review, we included RCTs that met the inclusion and exclusion criteria, as described in Textbox 1.

**Inclusion and exclusion criteria.**

**Inclusion criteria**

- Involved participants who were college or university students or young adults (aged 18-25 years, as defined by the Society for Adolescent Health and Medicine)
- Investigated the effects of sleep interventions delivered through electronic technologies, such as the internet, computers, tablets, mobile devices, telehealth, or virtual reality
- Included control groups comprising waiting-list control, placebo, no intervention, standard or usual care groups, or individuals receiving interventions delivered face-to-face through in-person coaching
- Assessed sleep outcomes of interest using either subjective measurements (eg, sleep questionnaires or sleep diaries) or objective measurements (eg, actigraphy)
- Reported both preintervention and postintervention outcome measures; no limitations regarding the language of publication were applied

**Exclusion criteria**

- Involved participants who were prescribed medication for sleep
- Involved participants who had been clinically diagnosed with severe sleep disorder (eg, obstructive sleep apnea, parasomnias, and narcolepsy), based on diagnostic criteria from the *Diagnostic and Statistical Manual of Mental Disorders, Fifth Edition*, and required more intensive treatment
- Involved participants who had psychiatric disorders (eg, major depressive disorder, bipolar disorder, or schizophrenia), diagnosed by a licensed clinician.

### Primary and Secondary Outcomes

The primary outcomes of this review were sleep quality, sleep parameters and insomnia severity. The secondary outcomes included dysfunctional beliefs and attitudes about sleep, sleep hygiene, sleep knowledge, and presleep arousal in both cognitive and somatic domains.

### Study Selection and Data Extraction

Duplicate articles were identified, removed, and screened using EndNote 20 (Clarivate) software. In total, 2 independent reviewers (YAL and HCL) screened the titles and abstracts of the remaining studies. Full-text reviews were conducted for studies meeting the inclusion criteria. Any disagreements between the 2 reviewers were resolved through discussion with a third reviewer (PST).

Data were extracted using Microsoft Excel with a standardized form by 2 independent reviewers (YAL and HCL). Information on the following parameters was collected: study characteristics (eg, first author, publication year, country, number of participants, mean age, number of female participants, and history of insomnia); intervention type (eg, duration and delivery mode); postintervention dropout rates; use of intention-to-treat analysis; comparison groups; and outcome measurements (eg, sleep quality and sleep parameters). Study authors were contacted if any data were missing or incomplete. Available data were used when no response was received from corresponding authors.

### Assessment of Methodological Quality

In total, 2 researchers (YAL and HCL) independently evaluated the quality of the included studies using the Cochrane Risk of Bias (version 2.0) tool for RCTs [35]. This tool is used to evaluate bias across the following five domains: (1) bias arising from the randomization process, (2) bias due to deviations from the intended intervention, (3) bias due to missing outcome data, (4) bias in the measurement of the outcome, and (5) bias in the selection of reported results. The overall risk of bias was determined by synthesizing findings across these domains, and studies were categorized as having a low risk of bias, some concerns, or a high risk of bias. Discrepancies between the researchers were resolved through discussion or, if necessary, consultation with a third reviewer (PST).

### Data Synthesis and Analysis

We conducted a meta-analysis of the pooled quantitative data using Comprehensive Meta-Analysis software (version 3.7; Biostat Inc). Effect sizes were calculated on the basis of reported means and SDs for each outcome. We analyzed preintervention to postintervention improvements and between-group differences in sleep-related outcomes. In addition, effect sizes for available follow-up measurements were calculated by comparing the intervention and control groups. A minimum of 2 studies per outcome was required to pool data for quantitative analyses.

A random-effects model was applied to account for anticipated heterogeneity across studies. The primary effect size, Hedges *g* was calculated for continuous outcomes and reported with 95% CIs [36]. Effect sizes were interpreted as small (0.2), moderate (0.5), or large (0.8) [37]. Heterogeneity was assessed using the *I*^2^ statistic, with values of 0%, 25%, 50%, and 75% indicating no, low, moderate, and high heterogeneity, respectively [38]. Substantial heterogeneity (ie, *I*^2^≥50%) was explored through moderator analyses and meta-regression. For outcomes with high heterogeneity and significant effects, we performed moderator analyses (for categorical variables) and meta-regression (for continuous variables) to identify potential sources of heterogeneity. Moderator analyses were limited to subgroups represented in at least 2 studies. A *P* value of <.05 was considered significant for both the moderator and the meta-regression analyses.

To ensure the robustness of our findings, sensitivity analyses were conducted by excluding studies with a high risk of bias and evaluating their effects on the overall results. High-risk studies were defined as those with severe systematic biases, such as unclear reporting of the randomization process, deviations from intended interventions, missing outcome data, problems with outcome measurements, or selective reporting of results [35]. Publication bias was assessed using Egger test and a funnel plot [39]. For outcomes reported in at least 10 studies, the trim-and-fill method was applied to estimate the potential impact of missing studies [40].

### Assessment of the Certainty of Evidence

The quality of evidence was evaluated using the Grading of Recommendations, Assessment, Development, and Evaluations framework. The quality of evidence was categorized as high, moderate, low, or very low on the basis of a comprehensive assessment of several factors, including risk of bias, inconsistency, indirectness, imprecision, and other relevant considerations [41].

## Results

### Study Selection

**Figure 1:**
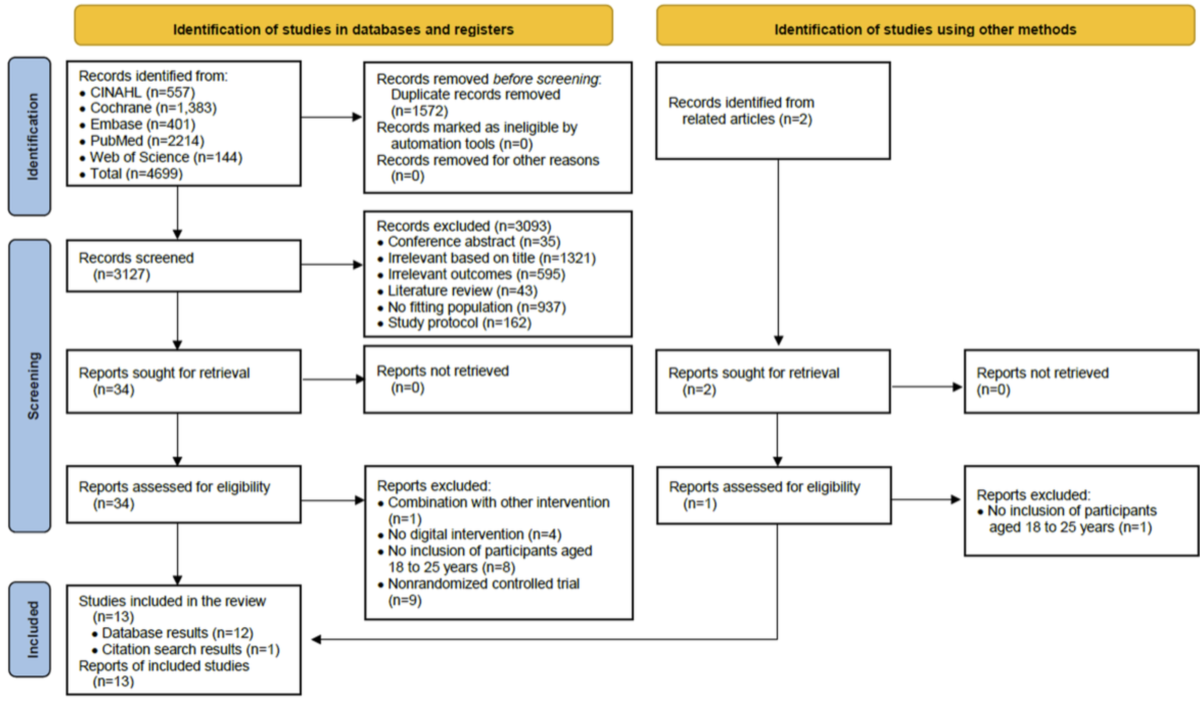
PRISMA (Preferred Reporting Items for Systematic Reviews and Meta-Analyses) flow diagram for study selection.

A total of 4699 records were identified through database searches, and 1572 duplicates were removed using EndNote 20. Two reviewers (YAL and HCL) independently dual-screened the titles and abstracts of the remaining studies. After the titles and abstracts were screened, 3093 records were deemed irrelevant and excluded. This process resulted in 34 studies being included for full-text review. Of these, 22 (65%) did not meet the inclusion criteria and were excluded from the systematic review. In addition, 1 study was identified through a manual search of reference lists in related articles. Finally, 13 (37%) studies were included in the meta-analysis [42-54]. The study selection process is summarized in Figure 1.

### Study Characteristics

The included studies involved 5251 college students and young adults, with a mean age of 23.6 (SD 3.16) years and 69.55% (3652/5251) of participants being women. Most studies were conducted in the United States (n=6, 46%), followed by the United Kingdom (n=3, 23%), Asia (n=3, 23%) and the Middle East (n=1, 8%). The sample sizes ranged from 40 to 3755 participants. The average dropout rate was 17.7% in the intervention group and 12.6% in the control group. Nearly 74.73% (3924/5251) of the participants reported sleep problems at baseline. The diagnostic criteria for assessing sleep problems included the Insomnia Severity Index (ISI) in 2 studies [52,53], the Sleep Condition Indicator (SCI) in 1 study [44], and the Sleep Quality Score in 1 study [49].

Most (11/13, 85%) of the studies used a 2-arm trial design, whereas 2 studies adopted a 3-arm trial design [48,49]. Jones et al [48] compared “text message–based sleep intervention” with “placebo” and “no intervention.” In addition, Kim et al [49] compared “virtual reality-based meditation” with “concentration meditation” and “no intervention.”

The effectiveness of digital sleep interventions was evaluated in terms of various outcomes: 13 studies assessed subjective sleep quality, 5 (38%) examined objective sleep parameters, 4 (31%) evaluated insomnia severity, 2 (15%) focused on dysfunctional beliefs and attitudes about sleep, 4 (31%) assessed sleep hygiene, 3 (23%) evaluated sleep knowledge, and 2 (15%) analyzed presleep arousal. The instruments used to assess subjective sleep quality included the Pittsburgh Sleep Quality Index, the Korean Modified Leeds Evaluation Questionnaire, the Patient Reported Outcomes Measurement Information System, and sleep diaries. Objective sleep parameters were assessed using actigraphy. Insomnia severity was evaluated using the ISI or SCI. Dysfunctional beliefs and attitudes about sleep were measured using the Dysfunctional Beliefs and Attitudes about Sleep-16 scale. Sleep hygiene and knowledge were assessed using the Sleep Hygiene Index or the Sleep Hygiene Practice Scale. Presleep arousal was measured using the Pre-Sleep Arousal Scale. The details of the included studies are presented in Table 1.

**Table 1:** Summary of characteristics of included studies.

Study, year, and country	Number of participants	Age (y), mean (SD)	Women, n (%)	History of insomnia	Dropout (AI^a^), %	Use of ITT^b^	Comparison	Outcome measurements
Barber and Cucalon [42], 2017, United States	IG^c^: 43 CG^d^: 35	IG: 20.67 (2.52) CG: 20.33 (1.66)	IG: 29 (37) CG: 18 (23)	No	IG: 0 CG: 0	Yes	Passive (waiting list)	Actigraphy^e^ SHI^f^ Sleep quality
Denis et al [43], 2020, United Kingdom	IG: 99 CG: 100	IG: 19.73 (2.94) CG: 20.22 (5.69)	IG: 99 (100) CG:100 (100)	No	IG: 32 CG: 22	No	Active (puzzles)	DBAS-16^g^ MCQ^h^ PSAS^i^ PSQI^j^ SCI^k^
Freeman et al [44], 2017, United Kingdom	IG: 1891 CG: 1864	IG: 24.83 (7.7) CG: 24.6 (7.6)	IG: 1361 (72) CG: 1315 (71)	Yes	IG: 59.1 CG: 38.7	No	Passive (usual practice)	ISI^l^ SCI Sleep diaries
Fucito et al [45], 2017, United States	IG: 21 CG: 21	IG: 24.83 (7.7) CG: 24.6 (7.6)	IG: 10 (48) CG: 10 (48)	No	IG: 9 CG: 9	Yes	Active (healthy behaviors)	Actigraphy^e^ PSQI PROMIS-SRI-SF^m^ Sleep diaries
Hershner and O’Brien [46], 2018, United States	IG: 254 CG: 295	IG: 21.9 (4.01) CG: 22 (4.35)	IG: 140 (55.8) CG: 176 (60.5)	No	IG: 42.9 CG: 30.2	No	Passive (no intervention)	ESS^n^ MEQ^o^ PSQI SHI
Huberty et al [47], 2019, United States	IG: 56 CG: 53	IG: 20.41 (2.31) CG: 21.85 (6.3)	IG: 36 (41) CG: 43 (49)	No	IG: 27 CG: 11	No	Passive (waiting list)	PROMIS^p^
Jones et al [48], 2020, United States	IG: 53 CG1: 52 CG2: 51	IG: 18.16 (0.37) CG1: 18.17 (0.38) CG2: 18.03 (0.17)	IG: 38 (79) CG1: 29 (69) CG2: 36 (80)	No	IG: 9 CG1: 19 CG2: 12	No	CG1: passive (Placebo);CG2: passive (no intervention)	PSQI SHI
Kim et al [49], 2024, Korea	IG: 20 CG1: 20 CG2: 20	IG: 21.6 (1.47) CG1: 21.75 (1.48) CG2: 22.35 (1.35)	IG: 15 (75) CG1: 13 (65) CG2: 13 (65)	Yes	IG: 0 CG1: 0 CG2: 0	Yes	CG1: active (Concentration meditation); CG2: passive (no intervention)	KMLSEQ-LSEQ^q^ Actigraphy^e^
Liu et al [50], 2024, Singapore	IG: 30 CG: 29	IG: 21.6 (4.26) CG: 22.4 (1.31)	IG: 12 (40) CG: 9 (31)	No	IG: 7 CG: 10	No	Active (sleep QA^r^ system)	Actigraphy^e^ PSQI Sleep diaries
Morris et al [51], 2016, United Kingdom	IG: 48 CG: 47	IG: 20.69 (2.61) CG: 20.27 (1.56)	IG: 29 (60) CG: 33 (70)	No	IG: 25 CG: 6	Yes	Passive (waiting list)	PSQI
Okajima et al [52], 2022, Japan	IG: 24 CG: 24	IG and CG: 19.56 (1.56)	IG: 16 (67) CG: 16 (67)	Yes	IG: 12 CG: 17	Yes	Passive (self-monitoring)	DBAS-16 FIRST^s^ ISI PSAS SHPS^t^ Sleep diaries
Short and Schmidt [53], 2020, United States	IG: 32 CG: 29	IG and CG: 19.43 (2.04)	IG and CG: 51 (84.0)	Yes	IG: 6 CG: 10	No	Active (physical health education)	ISI SRBQ^u^
Yıkılmaz et al [54], 2023, Turkey	IG: 20 CG: 20	IG: 20.7 (1.21) CG: 21.05 (0.94)	IG: 3 (15) CG: 2 (10)	No	IG: 0 CG: 0	Yes	Passive (no intervention)	PSQI

^a^AI: after intervention.

^b^ITT: intention-to-treat analysis.

^c^IG: intervention group.

^d^CG: control group.

^e^Objective measures.

^f^SHI: Sleep Hygiene Index.

^g^DBAS-16: Dysfunctional Beliefs And Attitudes About Sleep-16.

^h^MCQ: Munich Chronotype Questionnaire

^I^PSAS: Pre-Sleep Arousal Scale.

^j^PSQI: Pittsburgh Sleep Quality Index.

^k^SCI: Sleep Condition Indicator.

^l^ISI: Insomnia Severity Index.

^m^PROMIS-SRI-SF: Patient Reported Outcomes Measurement Information System Sleep-Related Impairment Short-Form.

^n^ESS: Epworth Sleepiness Scale.

^o^MEQ: Morningness-Eveningness Questionnaire.

^p^PROMIS: Patient Reported Outcomes Measurement Information System.

^q^KMLSEQ: Korean Modified Leeds Evaluation Questionnaire.

^r^QA: question answering.

^s^FIRST: Ford Insomnia Response to Stress Test.

^t^SHPS: Sleep Hygiene Practice Scale.

^u^SRBQ: Sleep-Related Behaviors Questionnaire.

### Quality Assessments

In total, 6 (46%) studies had a low overall risk of bias, 3 (23%) had a high overall risk of bias, and 4 (31%) had some concerns. Among the high-risk studies, 2 [42,46] did not report their randomization methods. In addition, one of these studies [46] had a high rate of missing data, compromising the integrity of the results because of its excessive data loss without explanation. Another study [47] was classified as high risk because it explicitly stated that blinding of participants and assessors was not feasible.

**Figure 2:**
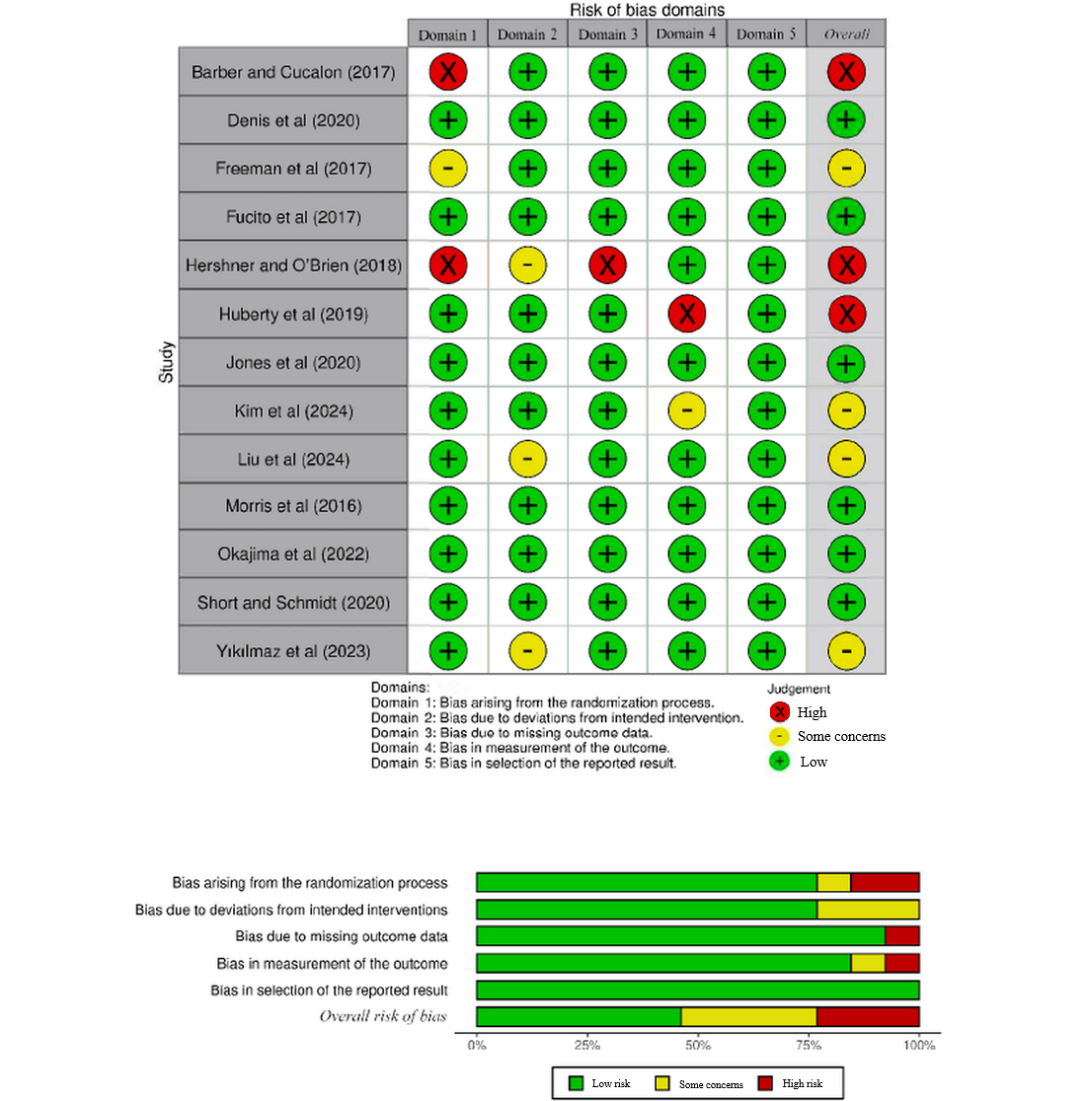
Risk of bias graph for included studies.

In total, 4 (31%) studies were categorized as having some concerns because of unclear information regarding specific domains. These included uncertainties about the randomization process [44], blinding of participants [50,54], and outcome measurement procedures [49]. All studies demonstrated a low risk of bias concerning selective reporting, and none provided sufficient information to evaluate other potential sources of bias. The details of the risk of bias assessment are illustrated in Figure 2 [42-54].

### Digital Sleep Interventions for College Students and Young Adults

The components of the digital sleep interventions varied across the included studies. Among the 13 studies, 5 (38%) used CBT-i, 5 (38%) focused on sleep-related education or information, 2 (15%) incorporated meditation, and 1 (8%) used body awareness therapy. The intervention durations ranged from 5 days to 10 weeks, with 4 studies [43-45,53] also assessing follow-up effects after the intervention period. Approximately half of the studies (n=7, 54%) were exclusively conducted in university settings. The detailed results are provided in Tables 1 and 2, and Table S1 in Multimedia Appendix 3 [42-54].

**Table 2:** Summary of digital intervention features of included studies.

Study, year, and country	Type	Duration	Mode of delivery
Barber and Cucalon [42], 2017, United States	Sleep hygiene	1 wk (22 min and 33 s)	Web (through Microsoft PowerPoint)
Denis et al [43], 2020, United Kingdom	Virtual therapist–led CBT-i^a^ (cognitive techniques, relaxation techniques, sleep hygiene, sleep restriction, and stimulus control)	6 wk (6 sessions per week, 20-25 min per session)	Web (through a video platform)
Freeman et al [44], 2017, United Kingdom	CBT-i (cognitive techniques, psychoeducation, relaxation techniques, sleep hygiene, sleep restriction, and stimulus control)	10 wk (6 sessions, average of 20 min per session)	Web
Fucito et al [45], 2017, United States	CBT-i (cognitive techniques, psychoeducation, relaxation techniques, sleep hygiene, and stimulus control)	4 wk (intervention consisted of 4 modules; 1 module per wk)	Web
Hershner and O’Brien [46], 2018, United States	Sleep education (a personality profile, 2 videos on sleep hygiene and its effects, and information on sleep behavior)	8 wk (intervention consisted of sleep modules of 20 min)	Web
Huberty et al [47], 2019, United States	Mindfulness meditation (body scan, breath focus, and kindness)	8 wk (at least 10 min per session)	Mobile app
Jones et al [48], 2020, United States	Health belief model education (theory based)	6 wk (2 texts [first 2 wk]; 1 text [second 2 wk]; and 1 text every other day [final 2 wk])	Online (through a text message)
Kim et al [49], 2024, Korea	Meditation (4 video types: sea, night sky, walking through a forest in the fall, and a green forest)	5 d (30 min per d)	VR^b^ (through a video)
Liu et al [50], 2024, Singapore	Sleep QA^c^ system with health coaching	4 wk (30 min per wk)	Online (through text)
Morris et al [51], 2016, United Kingdom	CBT-i (psychoeducation, relaxation techniques, sleep hygiene, sleep restriction, and stimulus control)	6 wk (intervention consisted of 7 modules; 20 min per wk)	Online (through text and email)
Okajima et al [52], 2022, Japan	CBT-i (cognitive techniques, relaxation techniques, sleep hygiene, sleep restriction, and stimulus control)	8 wk (30 min per session; 8 sessions per wk)	Online (through an email with PDF files)
Short and Schmidt [53], 2020, United States	Motivation, psychoeducation, behavioral tools, and behavior change modules	1 wk (intervention consisted of 4 modules of 45 min)	Online (through text)
Yıkılmaz et al [54], 2023, Turkey	Physiotherapist-led, internet-based BBAT^d^ intervention program	6 wk (60 min per session; 3 sessions per wk)	Videoconferencing

^a^CBT-i: cognitive behavioral therapy for insomnia.

^b^VR: virtual reality.

^c^QA: question answering.

^d^BBAT: basic body awareness therapy.

### Comparators

Most (n=10, 77%) of the included studies included a passive control group. In these studies, participants in the control group received usual or standard care, were placed on a waiting list, or did not receive any intervention. By contrast, the active control groups participated in sleep programs, received information on sleep education and healthy behaviors, or engaged in concentration meditation. The detailed results are provided in Table 1.

### Effectiveness of Digital Sleep Interventions

#### Sleep Quality

**Figure 3:**
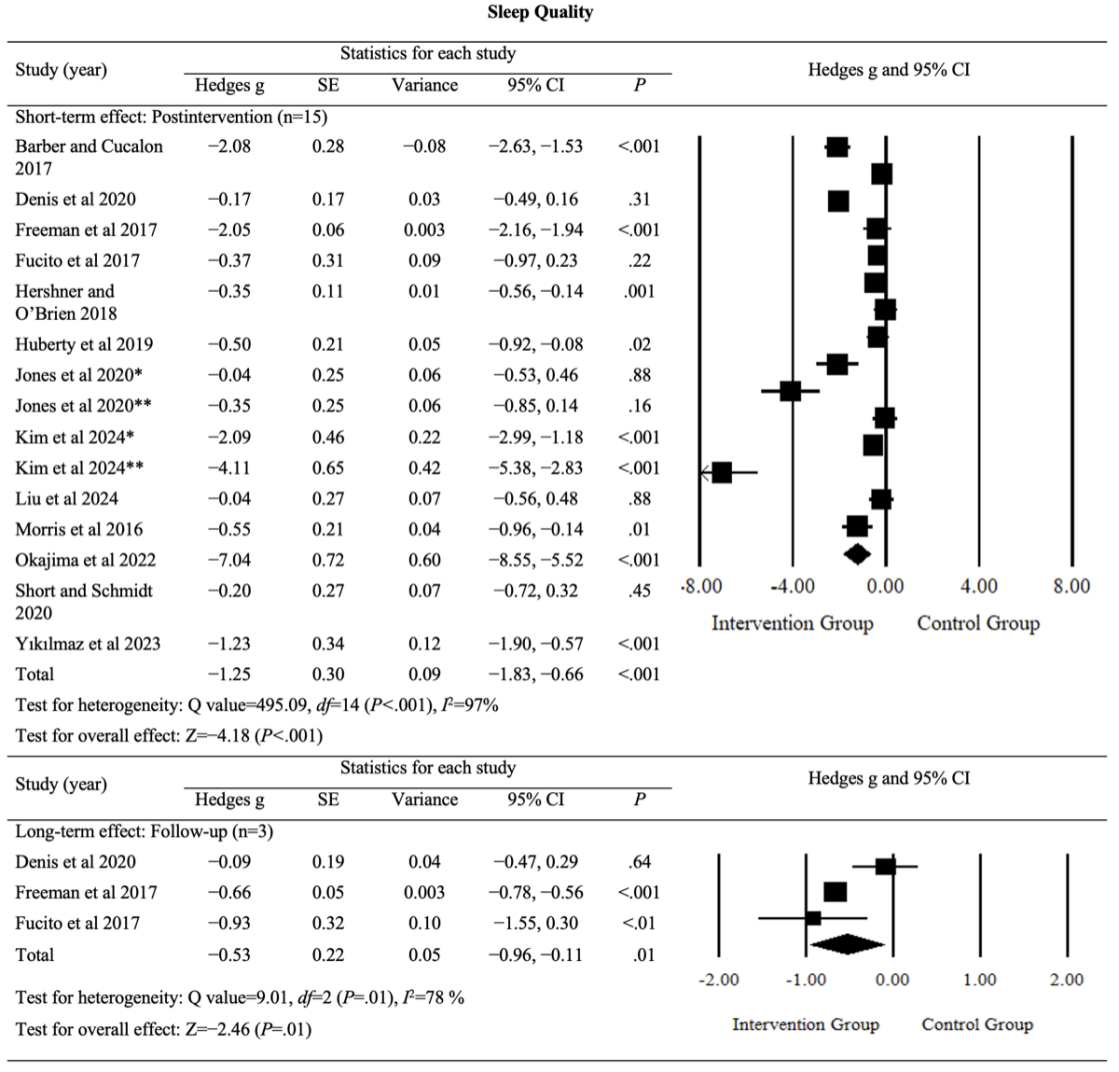
Effect sizes for outcomes of interest (sleep quality) in studies evaluating digital sleep interventions. *Digital intervention compared to active control. **Digital intervention compared to passive control.

The effects of digital sleep interventions on sleep quality were analyzed for both postintervention and follow-up periods. The pooled results revealed a large and significant postintervention effect (Hedges *g*=−1.25, 95% CI −1.83 to −0.66; *P*<.001; *I*^2^=97%) and a moderate but significant follow-up effect (Hedges *g*=−0.53, 95% CI −0.96 to −0.11; *P*=.01; *I*^2^=78%). Substantial heterogeneity was observed between the studies. The detailed results are provided in Figure 3 [42-54].

Although sleep quality is a critical outcome for college students and young adults, the certainty of evidence for the postintervention effects was rated as “low” (Table S1 in Multimedia Appendix 4). This rating was downgraded because of risks of bias, inconsistency, and imprecision but upgraded because of the large magnitude of the effect. For follow-up effects, the evidence was rated as having “moderate” certainty. These findings suggest that digital sleep interventions are likely to significantly improve sleep quality in both postintervention and follow-up assessments.

#### Sleep Parameters

**Figure 4:**
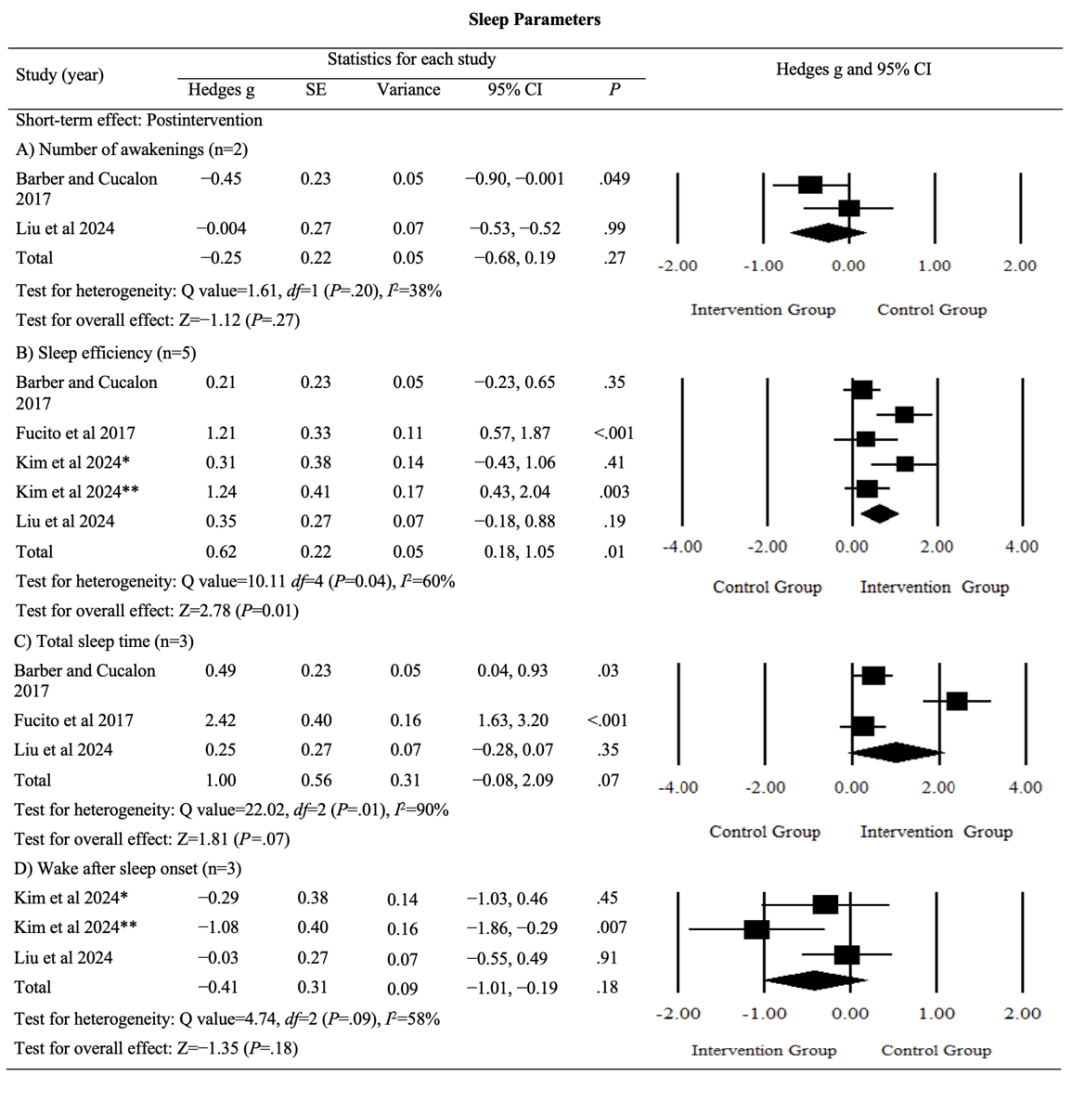
Effect sizes for outcomes of interest (sleep parameters) in studies evaluating digital sleep interventions. *Digital intervention compared to active control. **Digital intervention compared to passive control.

The pooled effects on sleep parameters, including sleep efficiency, total sleep time (TST), wake after sleep onset (WASO), and the number of awakenings (NWAK), were analyzed. Digital sleep interventions demonstrated a significant medium effect on sleep efficiency (Hedges *g*=0.62, 95% CI 0.18-1.05; *P*=.005; *I*^2^=60%), with substantial heterogeneity among studies. However, nonsignificant effects were observed for NWAK (*P*=.27), TST (*P*=.07), and WASO (*P*=.18). The detailed results are provided in Figure 4 [42,45,49,50].

Although sleep parameters are crucial outcomes, the certainty of the evidence ranged from “very low” to “low” (Table S1 in Multimedia Appendix 4). This low rating was because of increased risks of bias, inconsistency, and imprecision primarily resulting from variations in measurement methods for sleep parameters. Overall, the evidence remained highly uncertain regarding the postintervention effects of digital sleep interventions on sleep parameters.

#### Insomnia Severity

**Figure 5:**
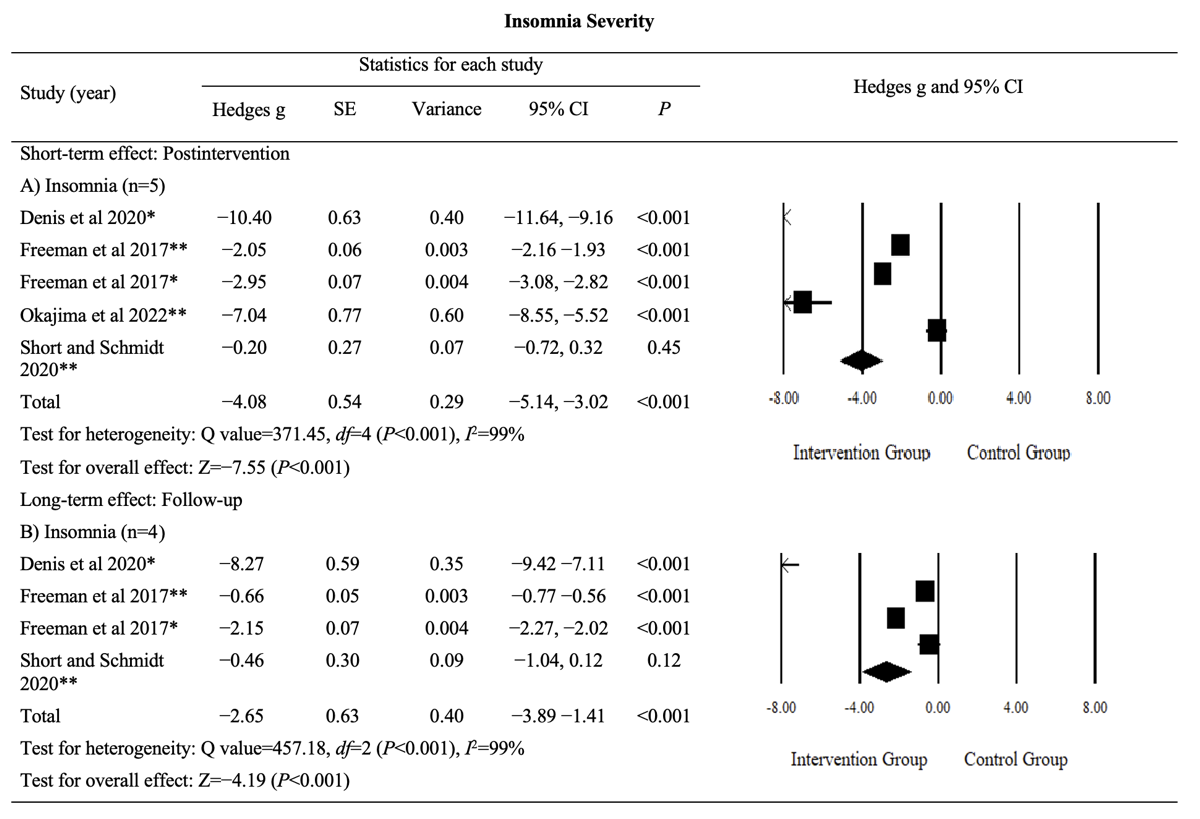
Effect sizes for outcomes of interest (insomnia severity) in studies evaluating digital sleep interventions. *Sleep Condition Indicator questionnaire. **Insomnia Severity Index questionnaire.

The pooled effects of digital sleep interventions on insomnia severity, as assessed using the ISI or SCI questionnaires, were analyzed for both postintervention and follow-up periods. The results indicated significant reductions in insomnia severity at postintervention (Hedges *g*=−4.08, 95% CI −5.14 to −3.02; *P*<.001; *I*^2^=99%) and follow-up assessments (Hedges *g*=−2.65, 95% CI −3.89 to −1.41; *P*<.001; *I*^2^=99%). Heterogeneity was observed in insomnia severity outcomes. The detailed results are provided in Figure 5 [43,44,52,53].

Insomnia severity is a critical outcome, and the evidence supporting the postintervention and follow-up effects of digital sleep interventions was rated as having a “moderate” level of certainty (Table S1 in Multimedia Appendix 4). This rating was downgraded because of inconsistency and imprecision but was then upgraded because of the large magnitude of the observed effects. These findings suggest that digital sleep interventions are likely to significantly reduce insomnia severity in both postintervention and follow-up assessments.

#### Dysfunctional Beliefs and Attitudes About Sleep

**Figure 6:**
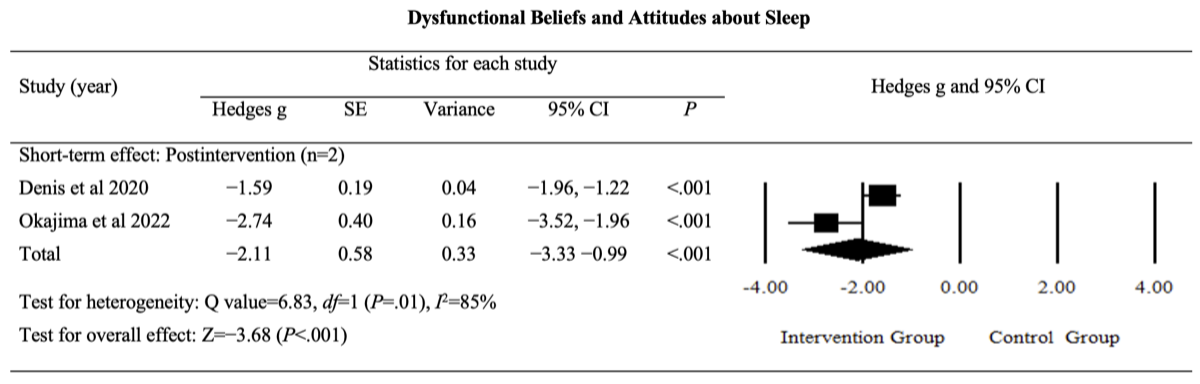
Effect sizes for outcomes of interest (dysfunctional beliefs and attitudes about sleep) in studies evaluating digital sleep interventions.

The pooled results of 2 studies reporting the postintervention effects of digital sleep interventions on dysfunctional beliefs and attitudes about sleep indicated a large and significant effect (Hedges *g*=−2.11, 95% CI −3.33 to −0.97; *P*<.001; *I*^2^=86%). The detailed results are provided in Figure 6 [43,52].

The certainty of evidence for this outcome was rated as “low,” reflecting the limited importance of the Dysfunctional Beliefs and Attitudes About Sleep-16 as a measure (Table S1 in Multimedia Appendix 4). This rating was downgraded due to inconsistency and imprecision in the findings. Although the evidence suggested that digital sleep interventions moderately affected dysfunctional beliefs and attitudes about sleep in postintervention assessments, the clinical significance of this outcome remained uncertain.

#### Sleep Hygiene

**Figure 7:**
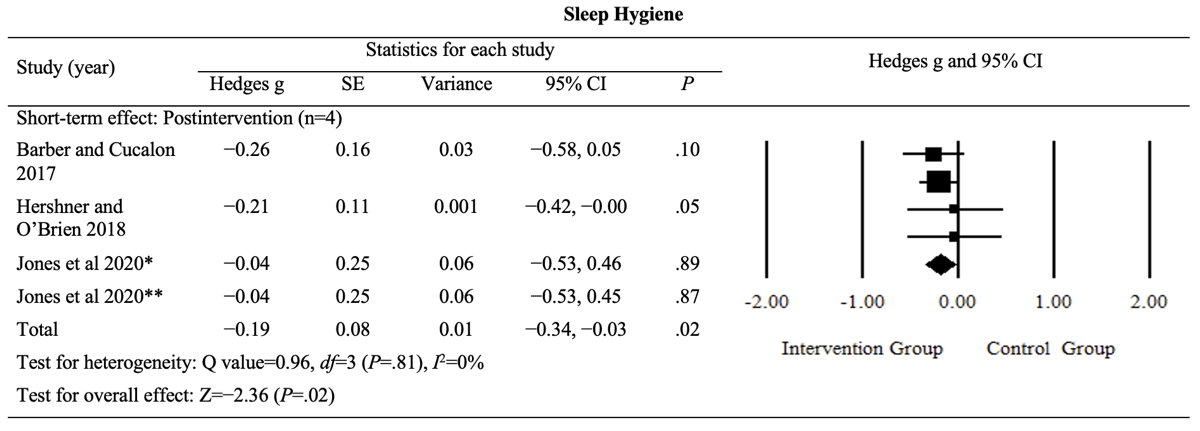
Effect sizes for outcomes of interest (sleep hygiene) in studies evaluating digital sleep interventions. *Digital intervention compared to active control. **Digital intervention compared to passive control.

Analysis of the pooled postintervention effects of digital sleep interventions on sleep hygiene revealed a small but significant improvement (Hedges *g*=−0.19, 95% CI −0.34 to −0.03; *P*=.02; *I*^2^=0%). The detailed results are provided in Figure 7 [42,46,48].

The evidence for this outcome was rated as having a “moderate” level of certainty, although sleep hygiene was considered an outcome of limited importance (Table S1 in Multimedia Appendix 4). The certainty rating was downgraded because of an increased risk of bias. These findings suggest that digital sleep interventions led to a slight improvement in sleep hygiene following the intervention.

#### Sleep Knowledge

**Figure 8:**
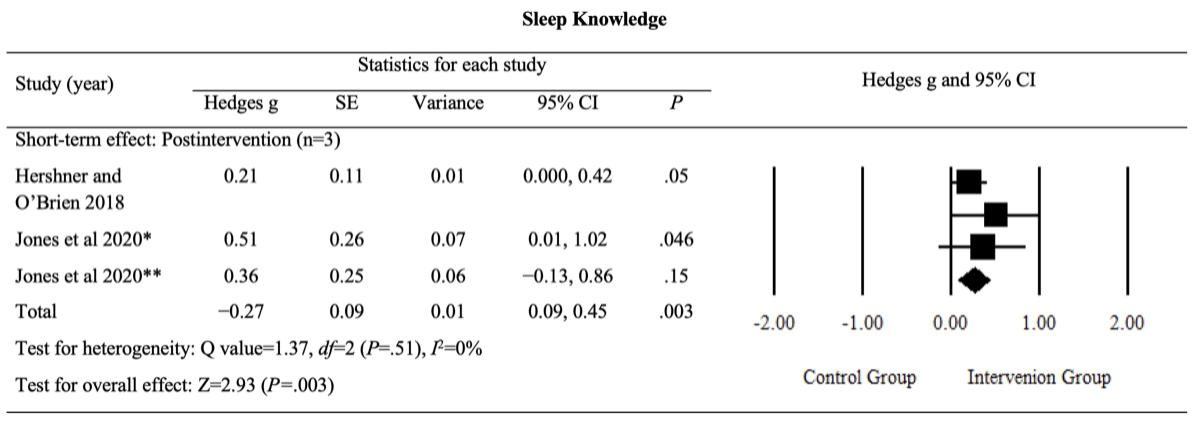
Effect sizes for outcomes of interest (sleep knowledge) in studies evaluating digital sleep interventions. *Digital intervention compared to active control. **Digital intervention compared to passive control.

The pooled results indicated a small but significant postintervention effect of digital sleep interventions on sleep knowledge (Hedges *g*=0.27, 95% CI 0.09-0.45; *P*=.003; *I*^2^=0%). The detailed results are provided in Figure 8 [46,48].

The evidence for this outcome was rated as having a “moderate” level of certainty, although sleep knowledge was considered an outcome of limited importance (Table S1 in Multimedia Appendix 4). The certainty rating was downgraded because of an increased risk of bias. These findings suggest that digital sleep interventions led to a slight improvement in sleep knowledge following the intervention.

#### Presleep Arousal

**Figure 9:**
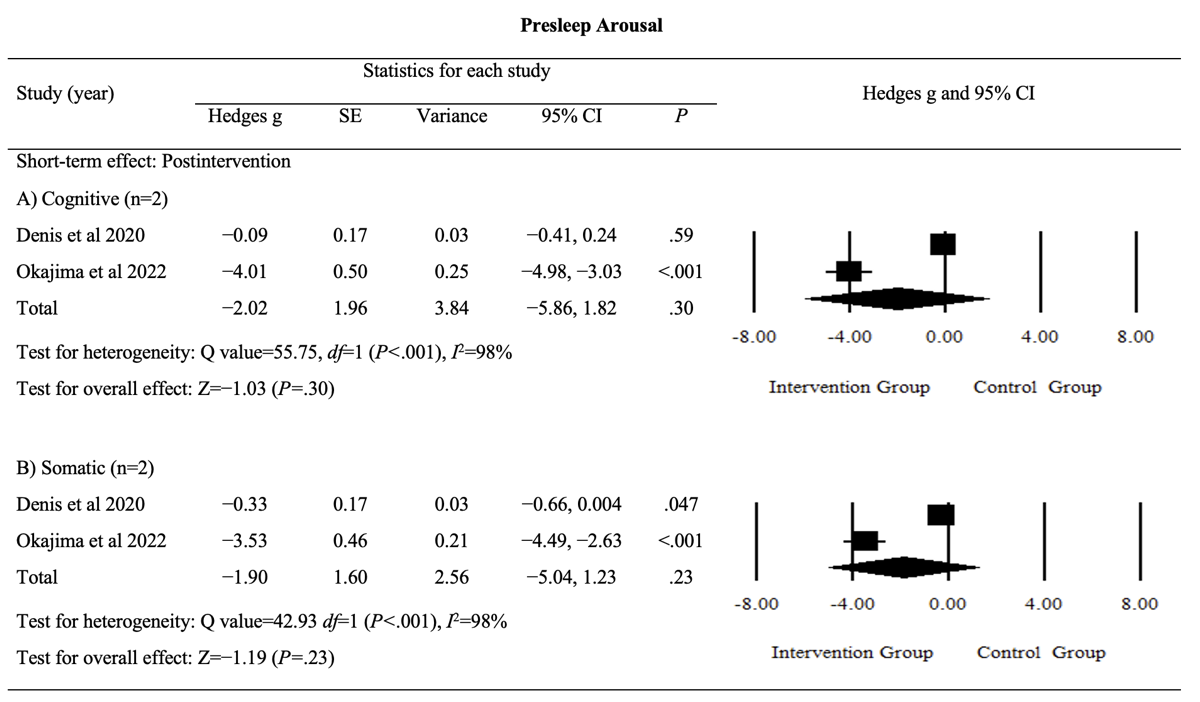
Effect sizes for outcomes of interest (presleep arousal) in studies evaluating digital sleep interventions.

The effects of digital sleep interventions on presleep arousal, including cognitive and somatic domains, were analyzed. The pooled results indicated nonsignificant postintervention effects for both the cognitive domain (Hedges *g*=−2.02, 95% CI −5.86 to 1.82; *P*=.30; *I*^2^=98%) and the somatic domain (Hedges *g*=−1.90, 95% CI −5.04 to 1.23; *P*=.23; *I*^2^=98%). The detailed results are provided in Figure 9 [43,52].

The certainty of evidence for these outcomes was rated as “low,” reflecting the limited importance of presleep arousal (cognitive and somatic domains) as a measure (Table S1 in Multimedia Appendix 4). The rating was downgraded because of inconsistency and imprecision in the findings. Overall, the evidence remained uncertain regarding the postintervention effects of digital sleep interventions on presleep arousal in both domains.

### Moderator Analysis

Moderator analysis identified several significant sources of heterogeneity in the effects of digital sleep interventions on sleep quality. Key factors included the type of sleep assessment tool used (PSQI vs other questionnaires; *P*<.001), intervention type (digital CBT-i vs other therapies; *P*<.001), intervention duration (≤6 wk vs >6 wk; *P*<.001), therapist guidance (guided vs unguided; *P*<.001), mode of delivery (email or text message vs video materials; *P*<.01), history of insomnia (yes vs no; *P*<.001), and the use of intention-to-treat analysis (yes vs no; *P*=.001).

In addition, heterogeneity in insomnia severity outcomes, both after intervention (ISI vs SCI; *P*=.001) and at follow-up (ISI vs SCI; *P*<.001), was primarily attributed to differences in the sleep assessment tool. Moreover, the variation in sleep efficiency was linked to intervention duration (1 vs 4 wk). The detailed results of the moderator (subgroup) analyses are provided in Table S1 in Multimedia Appendix 5.

### Meta-Regression Analysis

Meta-regression analyses indicated that neither mean age nor the percentage of women significantly contributed to heterogeneity in the effects of digital sleep interventions on various sleep-related outcomes (all *P*>.05).

Specifically, the effect size on sleep quality was not significantly associated with mean age (β=−.18, 95% CI −0.46 to 0.11; *P*=.23) or the percentage of women (β=.002, 95% CI −0.03 to 0.03; *P*=.90). Similarly, sleep efficiency was not significantly influenced by mean age (β=.06, 95% CI −0.55 to 0.68; *P*=.85) or the percentage of women (β=.003, 95% CI −0.04 to 0.04; *P*=.90).

The effect size on WASO also showed no significant association with mean age (β=−1.18, 95% CI −6.30 to 3.94; *P*=.65) or the percentage of women (β=−.02, 95% CI −.05 to .01; *P*=.12).

Similarly, insomnia severity, both after intervention and at follow-up, was not significantly associated with mean age (β=.31, 95% CI −0.39 to 1.01; *P*=.39 and β=.02, 95% CI −0.36 to 0.40; *P*=.90, respectively). The percentage of women also showed no significant association with insomnia severity postintervention (β=.13, 95% CI −0.02 to 0.27; *P*=.09) or at follow-up (β=.01, 95% CI −0.07 to 0.09; *P*=.85).

Finally, sleep efficiency was not significantly associated with mean age (β=−.03, 95% CI −0.15 to 0.08; *P*=.54) or the percentage of women (β=.01, 95% CI −0.02 to 0.04; *P*=.39).

### Sensitivity Analysis

Sensitivity analyses were conducted to assess the robustness of the findings of the meta-analysis. Exclusion of the data of 3 high-risk studies from the pooled data on sleep quality had a minimal impact on the results (*P*<.001), despite the substantial heterogeneity observed in postintervention sleep quality (*I*^2^=97%). In addition, a sensitivity analysis in which the data of one high-risk study were excluded from the pooled data on SE for sleep parameters revealed negligible changes in the results (*P*=.004), further supporting the robustness of the findings. The detailed results are provided in Table S2 in Multimedia Appendix 5.

### Publication Bias

Publication bias for sleep quality was assessed using the Egger test and a funnel plot. The Egger test indicated no significant publication bias (*P*=.24). The funnel plot is presented in Multimedia Appendix 6.

## Discussion

### Overview

This systematic review and meta-analysis evaluated the effects of digital sleep interventions on sleep-related outcomes in college students and young adults (aged 18 to 25 years). The findings demonstrate that digital sleep interventions significantly improved sleep quality, sleep efficiency as a sleep parameter, insomnia severity, dysfunctional beliefs and attitudes about sleep, sleep hygiene, and sleep knowledge. These results indicate the value of evidence-based digital sleep interventions for addressing sleep problems in this population.

### Sleep Quality and Insomnia Severity

Our results are consistent with those of previous studies [28,31] demonstrating that digital sleep interventions effectively alleviate sleep disturbances. Reviews have reported moderate-to-large effect sizes for improvements in sleep quality and reductions in insomnia severity. These results are particularly relevant given the high prevalence of poor sleep quality and insomnia among college students and young adults [55,56]. Digital interventions offer accessible and scalable solutions for managing sleep problems. This review noted that the beneficial effects of digital sleep interventions on sleep quality were sustained for 3 to 6 weeks after intervention, whereas improvements in insomnia severity persisted for up to 1 to 6 months. These findings highlight the potential of digital sleep interventions as a sustainable and effective treatment option for this demographic.

### Sleep Parameters

Digital sleep interventions significantly improved sleep efficiency but demonstrated limited effects on other sleep parameters. These findings are in line with those reported by Maurer et al [57] who examined the impact of digital CBT-i on sleep parameters in individuals with insomnia disorder. The observed improvements in sleep efficiency suggest that digital interventions can meaningfully enhance the proportion of time spent asleep while in bed. However, no significant effects were found for other objective measures, such as NWAK, TST, and WASO. This lack of consistency may be attributable to the variability in the methods used to measure sleep parameters across studies, which could have affected the outcomes [58]. Future research should prioritize standardizing sleep measures to improve consistency and reliability.

### Dysfunctional Beliefs and Attitudes About Sleep

Digital interventions effectively reduced dysfunctional beliefs and attitudes about sleep, consistent with findings reported by Thakral et al [59], who indicated the effectiveness of CBT-i in helping individuals develop healthier sleep-related beliefs and attitudes. However, this result contrasts with that reported by Linardon et al [60], whose meta-analysis found that smartphone app-based interventions for insomnia and sleep disturbances did not effectively reduce dysfunctional beliefs and attitudes about sleep. A potential explanation for this discrepancy is that the intervention in the study by Linardon et al [60] did not specifically address participants’ individual sleep-related beliefs and attitudes, which may have reduced the persuasiveness and impact of the CBT-i components. Our findings highlight the critical role of CBT-i as a core element in digital sleep interventions, given its demonstrated effectiveness in improving dysfunctional beliefs and attitudes about sleep outcomes [61-63].

### Sleep Hygiene and Sleep Knowledge

Although the improvements in sleep hygiene and sleep knowledge were smaller than those for other outcomes, they remain significant. This is consistent with findings reported by Bauducco et al [64] and Inhulsen et al [65] and by Chehri et al [66] who demonstrated that sleep hygiene practices are closely linked to sleep quality [64-66]. Promoting better sleep hygiene and increasing awareness of healthy sleep habits are essential strategies for addressing sleep problems. Sleep-related education that promotes knowledge and encourages behavioral changes is particularly effective at addressing sleep problems [67]. Such initiatives are especially valuable for raising awareness and establishing healthier sleep routines among college students and young adults (aged 18-25 years).

### Presleep Arousal

The effect sizes for Pre-Sleep Arousal Scale scores related to presleep arousal in both cognitive and somatic domains were nonsignificant, likely because the number of eligible RCTs was limited. Presleep arousal is a known predisposing factor for insomnia, with high levels in either domain commonly observed in individuals with sleep disorders, which considerably interferes with the ability to fall asleep [68,69]. Future interventions should incorporate additional components, such as mindfulness or relaxation techniques, to more effectively address this aspect of sleep disruption.

### Moderator

Moderator (subgroup) analyses revealed that digital CBT-i had greater effects on improving sleep quality than other digital sleep interventions, and therapist-guided interventions outperformed unguided ones. These findings suggest that therapist-guided digital CBT-i is a particularly effective approach to managing sleep problems [28]. Unlike the review by Hasan et al [28], which focused on general population, we incorporated both subjective and objective sleep data and specifically targeted college students and young adults. This focused approach provided deeper insights into the effectiveness of therapist-guided digital CBT-i in improving sleep outcomes for this population.

### Strengths and Limitations

To the best of our knowledge, this study is the first to comprehensively investigate the effects of various digital sleep interventions on sleep parameters among college students and young adults. By examining multiple sleep-related outcomes, we provided a holistic understanding of how digital interventions affect different aspects of sleep. However, several limitations of this study should be acknowledged. First, significant heterogeneity was observed across the studies due to variations in intervention regimens and outcome measures, which likely contributed to the inconsistencies in the results. Second, the inclusion of studies with a high risk of bias may have reduced the overall quality of evidence and affected the validity of the findings. However, sensitivity analyses in which high-risk studies were excluded revealed no significant changes in the outcomes, reinforcing the reliability of the results. Third, most studies relied heavily on self-reported measures, which are prone to biases, and objective assessments were underutilized. Finally, the lack of long-term follow-up data in many of the studies limited our ability to provide insights into the sustained impact of these interventions over time.

### Relevance for Implementation

We obtained robust evidence indicating that digital sleep interventions are an effective approach to addressing a variety of sleep problems among college students and young adults. These interventions offer a convenient and accessible method for managing sleep disturbances, serving as a low-intensity, cost-effective alternative for individuals who cannot or prefer not to access traditional sleep treatments. By helping users develop effective sleep-related skills, digital interventions can significantly improve sleep outcomes in this population. However, further research is required to enhance their effectiveness. Personalized digital tools that tailor interventions to individuals’ specific sleep patterns and behaviors should be explored. In addition, future RCTs should compare the effectiveness of guided versus unguided digital CBT-i, as therapist involvement may possibly exert attention effects and improve treatment adherence. Future research could benefit from incorporating qualitative approaches to gain deeper insight into user experiences with digital sleep interventions. In addition, combining both subjective and objective measures, such as actigraphy, would offer a more comprehensive evaluation of the interventions’ effectiveness. Longer follow-up periods are also required to enable assessment of the durability of intervention effects, ensuring these tools optimize their potential to improve sleep outcomes for college students and young adults.

### Conclusions

This review provides up-to-date evidence on the effectiveness of digital sleep interventions in improving various sleep-related outcomes among college students and young adults. According to the Grading of Recommendations Assessment, Development, and Evaluations assessment, the certainty of evidence ranged from moderate to very low. Specifically, moderate certainty was found for sleep quality, insomnia severity, sleep hygiene, and sleep knowledge, while other outcomes had low or very low certainty.

We conclude that digital sleep interventions can improve overall perceived sleep quality and insomnia severity in both postintervention and follow-up assessments. They may also have a slight impact on dysfunctional beliefs and attitudes about sleep, sleep hygiene, and sleep knowledge in postintervention assessments. However, the evidence remains highly uncertain regarding their effects on sleep parameters and somewhat uncertain concerning their impact on presleep arousal in both cognitive and somatic domains. These findings may be influenced by high heterogeneity, variations in interventions and outcome measures, reliance on self-reported data, and the absence of long-term follow-up, which limits the ability to assess the sustained effects of digital sleep interventions. Future research should aim to address these limitations to improve the applicability and effectiveness of digital sleep interventions.

## Appendix

Multimedia Appendix 1 PRISMA (Preferred Reporting Items for Systematic Reviews and Meta-Analyses) 2020 checklist.

Multimedia Appendix 2 Literature search strategy.

Multimedia Appendix 3 Detailed description of digital sleep interventions based on the Template for Intervention Description and Replication (TiDIER) checklist.

Multimedia Appendix 4 Grading of Recommendations Assessment, Development, and Evaluation (GRADE) summary.

Multimedia Appendix 5 Summary of moderator analysis and sensitivity analyses.

Multimedia Appendix 6 Funnel plot for publication bias.

## Abbreviations

CBT-i: cognitive behavioral therapy for insomnia
ISI: Insomnia Severity Index
NWAK: number of awakenings
PRISMA: Preferred Reporting Items for Systematic Reviews and Meta-Analyses
RCT: randomized controlled trial
SCI: Sleep Condition Indicator
TST: total sleep time
WASO: wake after sleep onset

## Data Availability

The datasets generated or analyzed during this study are available from the corresponding author on reasonable request
