# Supplementary material for: Effects of Digital Sleep Interventions on Sleep Among College Students and Young Adults: Systematic Review and Meta-Analysis
Source: J Med Internet Res. 2025 May 12;27:e69657. doi: 10.2196/69657 (PMC12107209; doi:10.2196/69657)
Supplement: Multimedia Appendix 2 [file jmir_v27i1e69657_app2.docx]

**Multimedia Appendix 2**

Table S1. Literature search strategy.

**Database: CINAHL**

|  | Searches | Results |
| --- | --- | --- |
| S1 | TI college OR TI university AND TI students OR TI young adults | 86,662 |
| S2 | AB digital OR AB sleep AND AB intervention | 50,595 |
| S3 | TI digital OR TI sleep AND TI intervention | 23,791 |
| S4 | S2 OR S3 | 64,511 |
| S5 | TX internet OR TX “web-based” OR TX online OR TX “e-mail” OR TX mobile OR TX smartphone OR TX “virtual reality” | 399,018 |
| S6 | S4 OR S5 | 450,604 |
| S7 | S1 AND S6 | 9,307 |
| S8 | TX sleep OR TX sleep quality OR TX insomnia | 119,512 |
| S9 | S7 AND S8 | 557 |

**Database: Cochrane Library**

|  | Searches | Results |
| --- | --- | --- |
| #1 | college OR university AND students OR “young adults” | 160,525 |
| #2 AND | digital OR sleep AND intervention OR Internet OR “web-based” OR online OR “e-mail” OR mobile OR smartphone OR “virtual reality” | 15,563 |
| #3 AND | sleep OR “sleep quality” OR insomnia | 2,235 |
| #4 AND | “randomized controlled trial” | 1,383 |

**Database: Embase**

|  | **Searches** | **Results** |
| --- | --- | --- |
| #1 | (college OR university) AND students OR “young adults” | 470,868 |
| #2 | (digital OR sleep) AND intervention | 60,186 |
| #3 | Internet OR “web-based” OR online OR “e-mail” OR mobile OR smartphone OR “virtual reality” | 1,178,384 |
| #4 | #2 OR #3 | 1,227,801 |
| #5 | sleep OR “sleep quality” OR insomnia | 551,076 |
| #6 | #1 AND #4 AND #5 | 4,043 |
| #7 | “randomized controlled trial” | 1,141,538 |
| #8 | #6 AND #7 | 401 |

Table S1. Literature search strategy (*continued*).

**Database: PubMed**

|  | **Searches** | **Results** |
| --- | --- | --- |
| #1 | (((college) OR (university)) AND (students)) OR ((young adults)) | 1,646,081 |
| #2 | ((digital) OR (sleep)) AND (intervention) | 551,211 |
| #3 | (((((((((digital) OR (sleep)) AND (intervention)) OR (internet)) OR (web-based)) OR (online)) OR (e-mail)) OR (mobile)) OR (smartphone)) OR (virtual reality) | 1,319,481 |
| #4 | ((sleep) OR (sleep quality)) OR (insomnia) | 323,290 |
| #5 | (((((college) OR (university)) AND (students)) OR ((young adults))) AND ((((((((((digital) OR (sleep)) AND (intervention)) OR (internet)) OR (web-based)) OR (online)) OR (e-mail)) OR (mobile)) OR (smartphone)) OR (virtual reality))) AND (((sleep) OR (sleep quality)) OR (insomnia)) | 21,515 |
| #6 | (((((college) OR (university)) AND (students)) OR ((young adults))) AND ((((((((((digital) OR (sleep)) AND (intervention)) OR (internet)) OR (web-based)) OR (online)) OR (e-mail)) OR (mobile)) OR (smartphone)) OR (virtual reality))) AND (((sleep) OR (sleep quality)) OR (insomnia)) Filters: Randomized Controlled Trial | 2,214 |

**Database: Web of Science**

|  | **Searches** | **Results** |
| --- | --- | --- |
| #1 | (((TI=(college)) OR TI=(university)) AND TI=(students)) OR TI=(‘young adults’) | 141,119 |
| #2 | (((TS=(college)) OR TS=(university)) AND TS=(students)) AND TS=(‘young adults’) | 12,268 |
| #3 | #1 OR #2 | 146,736 |
| #4 | ((ALL=(digital)) OR ALL=(sleep)) AND ALL=(intervention) | 67,876 |
| #5 | ((((((ALL=(internet)) OR ALL=(‘web-based’)) OR ALL=(online)) OR ALL=(‘e-mail’)) OR ALL=(mobile)) OR ALL=(smartphone)) OR ALL=(‘virtual reality’) | 1,489,667 |
| #6 | #4 OR #5 | 1,543,067 |
| #7 | ((ALL=(sleep)) OR ALL=(“sleep quality”)) OR ALL=(insomnia) | 410,255 |
| #8 | #6 AND #3 AND #7 | 1,716 |
| #9 | (#6 AND #3 AND #7) AND ALL=(‘randomized controlled trial’) | 144 |
