# Supplementary material for: Effects of Digital Sleep Interventions on Sleep Among College Students and Young Adults: Systematic Review and Meta-Analysis
Source: J Med Internet Res. 2025 May 12;27:e69657. doi: 10.2196/69657 (PMC12107209; doi:10.2196/69657)
Supplement: Multimedia Appendix 3 [file jmir_v27i1e69657_app3.docx]

**Multimedia Appendix 3**

Table S1. Detailed description of digital sleep interventions based on the Template for Intervention Description and Replication (TiDIER) checklist.

| No | Author, year | Brief Name | Why | What materials and procedures | Who provided | How | Where | When and how much | Tailoring |
| --- | --- | --- | --- | --- | --- | --- | --- | --- | --- |
| 1 | Baber and Cucalon [42], 2017 | Sleep treatment education program for students to include technology use (STEPS‐TECH) | Based on sleep hygiene education intervention. | 1. Automated PowerPoint; 2. written sleep diary; 3. actigraphy device | Unguided  (self-learning) | Web-based | U.S. Midwestern  university | 1 week  (watch PowerPoint for 22 min and 33 s) | Yes |
| 2 | Denis et al [43], 2020 | Sleepio program | Based on cognitive behavioral therapy for insomnia intervention. | 1. Web-based intervention; 2. digital cognitive behavioral therapy; 3. written sleep diary; 4. video materials | Guided by a virtual therapist | Web-based | Three universities in London | 6 weeks  (20–25 min/session/week) | No |
| 3 | Freeman et al [44], 2017 | Sleepio program | Based on cognitive behavioral therapy for insomnia intervention. | 1. Web-based intervention;  2. digital cognitive behavioral therapy; 3. email/text  message reminders; 4. written sleep diary | Unguided  (self-learning) | Web-based | 26 universities in the United Kingdom | 10 weeks  (6 sessions, average of 20 min/session) | No |
| 4 | Fucito et al [45], 2017 | Call it a Night (CIAN) | Based on cognitive behavioral therapy for insomnia intervention. | 1. Web-based intervention; 2. digital cognitive behavioral therapy; 3. email reminders; 4. actigraphy device | Unguided  (self-learning) | Web-based | Five New England colleges | 4 weeks  (intervention involving four modules) | No |
| 5 | Hershner and O’Brien [46], 2018 | Sleep to Stay Awake (sleeptostayawake.org) | Based on sleep education  intervention. | 1. Web-based intervention; 2. sleep education programs; 3. sleep personality profile | Unguided  (self-learning) | Web-based | Midwestern university | 8 weeks  (intervention involving sleep modules for 20 min) | Yes |

Table S1. Detailed description of digital sleep interventions based on the TiDIER checklist (*continued*).

| No | Author, year | Brief Name | Why | What materials and procedures | Who provided | How | Where | When and how much | Tailoring |
| --- | --- | --- | --- | --- | --- | --- | --- | --- | --- |
| 6 | Huberty et al [47], 2019 | Calm mobile app | Based on mindfulness meditation and integrated some cognitive behavioral therapy intervention | 1. Mobile apps-based; 2. mindfulness meditation program; 3. text reminders | Unguided  (self-learning) | Mobile apps | US  Southwestern university | 8 weeks  (at least 10 min/session) | No |
| 7 | Jones et al [48],  2020 | Research on freshman and sleeping habits  (REFRESH) study | Based on health belief model as a theoretical framework | 1. Web-based intervention; 2. sleep text messages; 3. text reminders | Unguided  (self-learning) | Web-based | Universities | 6 weeks  (2 texts [first 2 weeks]; 1 text [second 2 weeks];1 text every other day [final 2weeks]) | No |
| 8 | Kim et al [49],  2024 | Virtual reality–based meditation | Based on meditation and mindfulness meditation intervention | 1. Virtual reality–based; 2. mindfulness meditation program; 3. video materials;  4. actigraphy device | Guided by  a meditation instructor | Virtual Reality | Universities of  nursing in  Korea | 5 days  (4 video for 30 min each) | No |
| 9 | Liu et al [50],  2024 | Human–artificial intelligence (AI) sleep  coaching model | Based on large language models and medical information | 1. Internet-based intervention; 2. human–AI sleep coaching model with health coaching by peers; 3. written sleep diary; 4. actigraphy device | Guided by health coaching therapist | Web-based | University in Singapore | 4 weeks  (30 min/week) | Yes |
| 10 | Morris et al [51], 2016 | Internet-delivered cognitive behavioral therapy | Based on classical cognitive behavioral therapy for insomnia intervention | 1. Internet-based intervention; 2. digital cognitive behavioral therapy through self-help programs; 3. email/text reminders | Unguided  (self-learning) | Web-based | University of Bristol in United Kingdom | 6 weeks  (intervention involving seven modules for 20 min/week) | No |

Table S1. Detailed description of digital sleep interventions based on the TiDIER checklist (*continued*).

| No | Author, year | Brief Name | Why | What materials and procedures | Who provided | How | Where | When and how much | Tailoring |
| --- | --- | --- | --- | --- | --- | --- | --- | --- | --- |
| 11 | Okajima et al [52], 2022 | Email-delivered cognitive behavioral therapy for insomnia (REFRESH) | Based on cognitive behavioral therapy for insomnia intervention | 1. Web-based intervention; 2. email messages with  attached PDF files; 3. written sleep diary; 4. homework | Unguided  (self-learning) | Web-based | A university campus in Japan | 8 weeks  (30 min for each session; 8 sessions/week) | No |
| 12 | Short and Schmidt [53], 2020 | Computerized cognitive behavioral  intervention | Based on false safety  aid elimination therapy  (FEST) | 1. Internet-based intervention; 2. sleep treatment program | Unguided  (self-learning) | Web-based | A university in the United States | 1 week  (intervention involving four modules of 45 min each) | No |
| 13 | Yıkılmaz et al [54], 2023 | Telerehabilitation-based basic body awareness therapy (BBAT) | No information | 1. Internet-based BBAT intervention; 2. exercise programs; 3. zoom software | Guided by a physiotherapist | Videoconferencing | A university in  Turkey | 6 weeks  (60 min for each session; 3 sessions/week) | Yes |
