# Supplementary material for: Effects of Digital Sleep Interventions on Sleep Among College Students and Young Adults: Systematic Review and Meta-Analysis
Source: J Med Internet Res. 2025 May 12;27:e69657. doi: 10.2196/69657 (PMC12107209; doi:10.2196/69657)
Supplement: Multimedia Appendix 4 [file jmir_v27i1e69657_app4.docx]

**Multimedia Appendix 4**

Table S1. Grading of Recommendations Assessment, Development, and Evaluation (GRADE) summary.

| Quality of evidence | | | | | | | | No. of patients (IG/CG) | Effect | | | Certainty of evidence |
| --- | --- | --- | --- | --- | --- | --- | --- | --- | --- | --- | --- | --- |
| No. of studies | Study design | Risk of bias | Inconsistency | Indirectness | Imprecision | Publication bias | Other considerations |  | Effect size  (Hedges’ g) | 95% CI  (Lower, upper limit) | *I*^2^ |  |
| **1. Sleep Quality** | | | | | | | | | | | | |
| *1.1 Postintervention effect* ^a^ | | | | | | | | | | | | |
| 13 | RCTs | Serious | Serious | Not serious | Serious | Not detected | None | 2,591/2,560 | −1.27 | −1.85, −0.65 | 97 % | ⨁⨁◯◯  Low |
| *1..2 Follow-up effect* ^b^ | | | | | | | | | | | | |
| 5 | RCTs | Not Serious | Serious | Not serious | Not Serious | Not detected | None | 2,011/1,985 | −0.53 | −0.96, −0.11 | 78 % | ⨁⨁⨁◯  Moderate |
| **2. Sleep Parameters** ^a^ | | | | | | | | | | | | |
| *2-1. Number of awakenings* | | | | | | | | | | | | |
| 2 | RCTs | Not Serious | Serious | Not serious | Serious | NA | None | 73/64 | −0.25 | −0.68, 0.19 | 38 % | ⨁⨁◯◯  Low |
| *2-2. Sleep efficiency* | | | | | | | | | | | | |
| 5 | RCTs | Serious | Serious | Not serious | Not Serious | Not detected | None | 114/125 | 0.62 | 0.18, 1.05 | 60% | ⨁⨁◯◯  Low |
| *2-3. Total sleep time* | | | | | | | | | | | | |
| 3 | RCTs | Serious | Serious | Not serious | Serious | NA | None | 94/85 | 1.00 | −0.08, 2.09 | 91 % | ⨁◯◯◯  Very Low |
| *2-4. Wake after sleep onset* | | | | | | | | | | | | |
| 2 | RCTs | Not Serious | Serious | Not serious | Serious | NA | None | 50/69 | −0.41 | −1.01, −0.19 | 58% | ⨁⨁◯◯  Low |

Table S1.Grading of Recommendations Assessment, Development, and Evaluation (GRADE) summary (*continued*).

| Quality of evidence | | | | | | | | No. of patients (IG/CG) | Effect | | | Certainty of evidence |
| --- | --- | --- | --- | --- | --- | --- | --- | --- | --- | --- | --- | --- |
| No. of studies | Study design | Risk of bias | Inconsistency | Indirectness | Imprecision | Publication bias | Other considerations |  | Effect size  (Hedges’ g) | 95% CI  (Lower, upper limit) | *I*^2^ |  |
| **3. Insomnia Severity** | | | | | | | | | | | | |
| *3.1 Postintervention effect* ^a^ | | | | | | | | | | | | |
| 4 | RCTs | Not Serious | Serious | Not serious | Serious | Not detected | None | 2,064/2,017 | −4.08 | −5.14, −3.02 | 99% | ⨁⨁⨁◯  Moderate |
| *3.2 Follow-up effect* ^b^ | | | | | | | | | | | | |
| 3 | RCTs | Not Serious | Serious | Not serious | Serious | NA | None | 2,064/1,993 | −2.65 | −3.89, −1.41 | 99% | ⨁⨁⨁◯  Moderate |
| **4. Dysfunctional Beliefs and Attitudes about Sleep** ^a^ | | | | | | | | | | | | |
| 2 | RCTs | Not serious | Serious | Not serious | Serious | NA | None | 123/124 | −2.11 | −3.33, −0.99 | 85% | ⨁⨁◯◯  Low |
| **5. Sleep Hygiene** ^a^ | | | | | | | | | | | | |
| 4 | RCTs | Serious | Not serious | Not serious | Not serious | Not detected | None | 350/433 | −0.19 | −0.34, −0.03 | 0% | ⨁⨁⨁◯  Moderate |
| **6. Sleep Knowledge** ^a^ | | | | | | | | | | | | |
| 3 | RCTs | Serious | Not serious | Not serious | Not serious | Not detected | None | 307/398 | −0.27 | 0.09, 0.45 | 0% | ⨁⨁⨁◯  Moderate |
| **7. Presleep Arousal** ^a^ | | | | | | | | | | | | |
| *7.1 Cognitive domain* | | | | | | | | | | | | |
| 2 | RCTs | Not serious | Serious | Not serious | Serious | NA | None | 123/124 | −2.02 | −5.86, 1.82 | 98% | ⨁⨁◯◯  Low |
| *7.2 Somatic domain* | | | | | | | | | | | | |
| 2 | RCTs | Not serious | Serious | Not serious | Serious | NA | None | 123/124 | −1.90 | −5.04, 1.23 | 98% | ⨁⨁◯◯  Low |

Note: CG=control group, CI=confidence interval, IG=intervention group, NA=not applicable, RCTs=randomized controlled trials, ^a^ Postintervention effect, ^b^ Follow-up effect
