# Supplementary material for: Effects of Digital Sleep Interventions on Sleep Among College Students and Young Adults: Systematic Review and Meta-Analysis
Source: J Med Internet Res. 2025 May 12;27:e69657. doi: 10.2196/69657 (PMC12107209; doi:10.2196/69657)
Supplement: Multimedia Appendix 5 [file jmir_v27i1e69657_app5.docx]

**Multimedia Appendix 5**

Table S1. Summary of moderator analysis (subgroup analysis).

| Group | No. of RCTs | Hedges’ g | Standard error | Variance | 95% CI | *P* value | |
| --- | --- | --- | --- | --- | --- | --- | --- |
|  |  |  |  |  |  | Each category | Between group |
| **1. Tool of sleep assessment** |  |  |  |  |  |  |  |
| ***1-1. Sleep quality*** |  |  |  |  |  |  | < .001 |
| *[Postintervention effect]* |  |  |  |  |  |  |  |
| PSQI questionnaire | 8 | −0.34 | 0.10 | 0.01 | −0.54, −0.15 | < .001 |  |
| Other questionnaires | 7 | −2.35 | 0.47 | 0.22 | −3.27, −1.43 | < .001 |  |
| ***1-2A. Insomnia severity*** |  |  |  |  |  |  | .001 |
| *[Postintervention effect]* |  |  |  |  |  |  |  |
| ISI questionnaire | 3 | −2.96 | 0.99 | 0.97 | −4.90, −1.03 | .003 |  |
| SCI questionnaire | 2 | 0.56 | 0.20 | 0.04 | 0.16, 0.95 | .006 |  |
| ***1-2B. Insomnia severity*** |  |  |  |  |  |  | < .001 |
| *[**Follow-up effect]* |  |  |  |  |  |  |  |
| ISI questionnaire | 2 | −0.66 | 0.05 | 0.003 | −0.76, −0.53 | < .001 |  |
| SCI questionnaire | 2 | 0.56 | 0.18 | 0.03 | 0.20, 0.92 | .002 |  |
|  | | | | | | | |
| **2. Intervention duration** |  |  |  |  |  |  |  |
| ***2-1. Sleep quality*** |  |  |  |  |  |  | < .001 |
| *[Postintervention effect]* |  |  |  |  |  |  |  |
| ≤ 6 weeks | 6 | −1.38 | 0.50 | 0.25 | −2.37, −0.40 | .006 |  |
| > 6 weeks | 9 | −1.18 | 0.39 | 0.15 | −1.94, −0.41 | .003 |  |
| ***2-2A. Insomnia severity*** |  |  |  |  |  |  | .93 |
| *[Postintervention effect]* |  |  |  |  |  |  |  |
| ≤ 6 weeks | 2 | 0.10 | 0.26 | 0.07 | −0.41, 0.61 | .70 |  |
| > 6 weeks | 3 | −2.54 | 1.18 | 1.40 | −4.92, −0.27 | .03 |  |
| ***2-2B. Insomnia severity*** |  |  |  |  |  |  | .95 |
| *[Follow-up effect]* |  |  |  |  |  |  |  |
| ≤ 6 weeks | 2 | −0.03 | 0.39 | 0.15 | −0.08,0.73 | .93 |  |
| > 6 weeks | 2 | 0.02 | 0.68 | 0.47 | −1.37,1.36 | .98 |  |
| ***2-3.*** ***Sleep efficiency*** |  |  |  |  |  |  | .02 |
| *[Postintervention effect]* |  |  |  |  |  |  |  |
| 1 week | 3 | 0.52 | 0.30 | 0.09 | −0.07, 0.11 | .08 |  |
| 4 weeks | 2 | 0.76 | 0.44 | 0.19 | −0.09, 1.62 | .08 |  |

Table S1. Summary of moderator analysis (subgroup analysis) (*continued*).

| Group | No. of RCTs | Hedges’ g | Standard error | Variance | 95% CI | *P* value | |
| --- | --- | --- | --- | --- | --- | --- | --- |
|  |  |  |  |  |  | Each category | Between group |
| **3. Type of intervention** |  |  |  |  |  |  |  |
| ***3-1. Sleep quality*** |  |  |  |  |  |  | < .001 |
| *[Postintervention effect]* |  |  |  |  |  |  |  |
| Digital CBT-i therapy | 5 | −1.79 | 0.61 | 0.37 | −2.98, −0.60 | .003 |  |
| Other therapies | 10 | −0.95 | 0.25 | 0.06 | −1.44, −0.46 | < .001 |  |
|  | | | | | | | |
| **4 Therapist**-**provided instructions** |  |  |  |  |  |  |  |
| ***1. Sleep quality*** |  |  |  |  |  |  | < .001 |
| *[Postintervention effect]* |  |  |  |  |  |  |  |
| Guided | 5 | −1.39 | 0.52 | 0.27 | −2.41, −0.37 | .008 |  |
| Unguided | 10 | −1.19 | 0.37 | 0.14 | −1.91, −0.46 | .001 |  |
|  | | | | | | | |
| **5. Mode of delivery** |  |  |  |  |  |  |  |
| ***1. Sleep quality*** |  |  |  |  |  |  | .002 |
| *[Postintervention effect]* |  |  |  |  |  |  |  |
| Email/Text message | 6 | −1.06 | 0.45 | 0.20 | −1.94, −0.17 | .02 |  |
| Video materials | 3 | −2.05 | 1.13 | 1.27 | −4.27, 0.16 | .07 |  |
|  | | | | | | | |
| **6. History of insomnia** |  |  |  |  |  |  |  |
| ***1. Sleep quality*** |  |  |  |  |  |  | < .001 |
| *[Postintervention effect]* |  |  |  |  |  |  |  |
| Yes | 5 | −2.91 | 0.70 | 0.49 | −4.28, −1.55 | < .001 |  |
| No | 10 | −0.54 | 0.16 | 0.03 | −0.85, −0.23 | .001 |  |
|  | | | | | | | |
| **7. ITT application** |  |  |  |  |  |  |  |
| ***1. Sleep quality*** |  |  |  |  |  |  | .001 |
| *[Postintervention effect]* |  |  |  |  |  |  |  |
| Yes | 7 | −2.33 | 0.57 | 0.32 | −3.44, −1.22 | < .001 |  |
| No | 8 | −0.47 | 0.40 | 0.16 | −1.26, −0.31 | .24 |  |

Note: CBT-i=cognitive behavioral therapy for insomnia, CI=confidence interval, ITT=intention-to-treat, ISI=insomnia severity index,

PSQI=Pittsburgh sleep quality index, RCTs=randomized controlled trials, SCI=sleep condition indicator

Table S2. Sensitivity analyses for outcomes of interest.

| Outcomes | No. of studies | *I*^2^ | Hedges’ g | Standard error | 95% CI | *P* value |
| --- | --- | --- | --- | --- | --- | --- |
| **Sleep quality** |  |  |  |  |  |  |
| Excluded high-risk studies | 12 | 97 % | −1.37 | 0.36 | −2.07, −0.66 | < .001 |
| **Sleep parameters:** |  |  |  |  |  |  |
| ***Sleep efficiency*** |  |  |  |  |  |  |
| Excluded high-risk studies | 4 | 57 % | 0.75 | 0.26 | 0.25, 1.26 | .004 |

Note: CI=confidence interval
