# Supplementary figures and images for: Effects of Digital Sleep Interventions on Sleep Among College Students and Young Adults: Systematic Review and Meta-Analysis
Source: J Med Internet Res. 2025 May 12;27:e69657. doi: 10.2196/69657 (PMC12107209; doi:10.2196/69657)

**Multimedia Appendix 6**


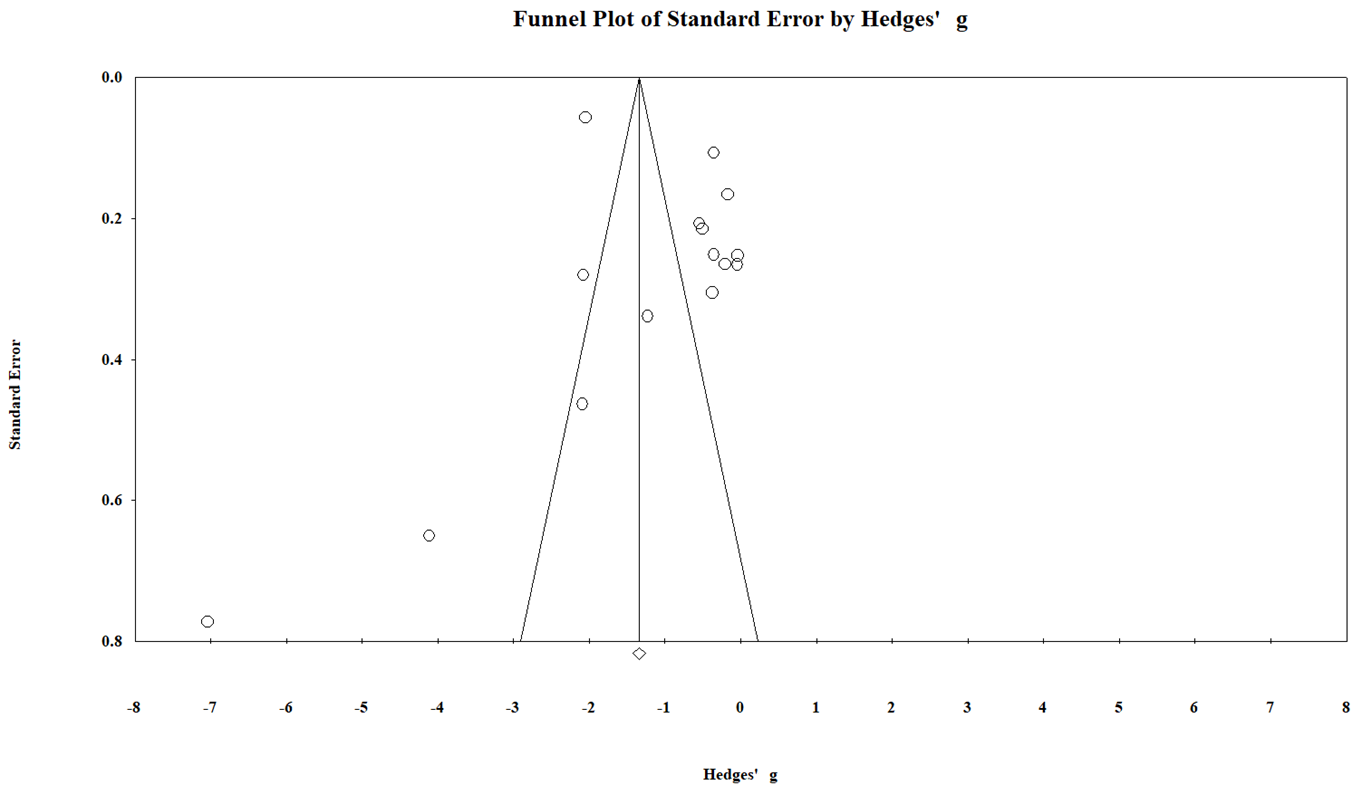


Funnel plot for publication bias

Egger’s test results: t = 1.22 and df = 13, *P* = .24

Supplement: Multimedia Appendix 6 [file jmir_v27i1e69657_app6.docx]
